# 3D Bioprinting of Smart Oxygen-Releasing Cartilage Scaffolds

**DOI:** 10.3390/jfb13040252

**Published:** 2022-11-17

**Authors:** Caterine Yesenia Carrasco Montesdeoca, Thiago Domingues Stocco, Fernanda Roberta Marciano, Thomas J. Webster, Anderson Oliveira Lobo

**Affiliations:** 1 Faculty of Medical Science, State University of Campinas, Campinas 13083-872, SP, Brazil; 2 School of Biological Sciences and Engineering, Yachay Tech University, Urcuquí 100650, Ecuador; 3 Interdisciplinary Laboratory for Advanced Materials (LIMAV), Materials Science and Engineering Graduate Program (PPGCM), Federal University of Piaui (UFPI), Teresina 64049-550, PI, Brazil; 4 Bioengineering Program, Scientific and Technological Institute, Brasil University, São Paulo 13566-590, SP, Brazil; 5 Department of Physics, UFPI—Federal University of Piaui, Teresina 64049-550, PI, Brazil; 6 School of Engineering, Saveetha University, Chennai 602117, India; 7 School of Health Sciences and Biomedical Engineering, Hebei University of Technology, Tianjin 065000, China

**Keywords:** 3D bioprinting, smart biomaterials, cartilage tissue regeneration, gelatin methacryloyl (GelMA), oxygen-releasing nanoparticles

## Abstract

Three-dimensional bioprinting is a powerful technique for manufacturing improved engineered tissues. Three-dimensional bioprinted hydrogels have significantly advanced the medical field to repair cartilage tissue, allowing for such constructs to be loaded with different components, such as cells, nanoparticles, and/or drugs. Cartilage, as an avascular tissue, presents extreme difficulty in self-repair when it has been damaged. In this way, hydrogels with optimal chemical and physical properties have been researched to respond to external stimuli and release various bioactive agents to further promote a desired tissue response. For instance, methacryloyl gelatin (GelMA) is a type of modified hydrogel that allows for the encapsulation of cells, as well as oxygen-releasing nanoparticles that, in the presence of an aqueous medium and through controlled porosity and swelling, allow for internal and external environmental exchanges. This review explores the 3D bioprinting of hydrogels, with a particular focus on GelMA hydrogels, to repair cartilage tissue. Recent advances and future perspectives are described.

## 1. Introduction

Osteoarthritis (OA) results from inflammatory joint diseases associated with a sedentary lifestyle, physical inactivity, and obesity that contribute to metabolic changes. The cartilage from patients with OA at a later stage of the disease does not seem to have this natural metabolic flexibility [1]. Cartilage tissue is one of the most critical tissues in need of repair. It cannot regenerate itself because of the small amount of constituent oxygen, due to a lack of blood vessels, nerves, and lymphatic vessels, which form a hypoxic environment [2]. Thus, cartilage cells do not have sufficient oxygen to proliferate and differentiate, causing cell death after injury, only receives nutrients from subchondral bone and the synovial fluid. Approximately 3.2% of cartilage is an avascular zone, compared to the proliferative zone, which is highly vascularized and presents approximately 7.5% oxygen [2,3,4]. A period of 4–6 weeks is required to reach complete vascularity, with an 83% patency for damaged tissue [5].

Cartilage tissue regeneration takes 21 days to regenerate completely, and the most critical period is after a cartilage-regenerating material is injected, requiring oxygen supplementation [3,6]. To help, tissue engineering has developed biological materials (biomaterials) for cartilage regeneration based on hydrogels made of natural and/or synthetic polymers with compositional and mechanical properties, comparable to the native extracellular matrix of cartilage. A major problem with traditional scaffolds is that they are unable to form an environment with adequate concentrations of oxygen inside, thus impairing potential cartilage tissue regeneration [7]. Some technologies have been constructed to supply oxygen and promote cell growth for cartilage applications, such as perfusion bioreactors, microfluidic techniques, and the incorporation of scaffolding matrices formed by angiogenic cells for rapid neovascularization and to ease blood vessel formation. However, excellent results have not been achieved, due to the inhomogeneity of 3D growth factors, the poor vasculature formed both in vitro and in vivo, and the lack of oxygen [5].

In recent years, different natural and synthetic materials, such as gelatin, chitosan, chondroitin sulfate, hyaluronic acid, poly (vinyl alcohol) (PVA), and methacryloyl gelatin (GelMA), have been used to fabricate biomaterials for cartilage repair [8]. Biomaterials supplying oxygen have been developed by loading oxygen-releasing particles inside hydrogels, which break down in an aqueous medium, forming functional biological scaffolds. Some materials, shaped like particles, that release oxygen have been studied, including perfluorocarbon (PFC) emulsions [9], hemoglobin particles [10], calcium peroxide (CPO), hydrogen peroxide (H_2_O_2_), and sodium percarbonate particles (SPO) [11], which can form oxygen inside, or next to, the created tissues to foster cell migration, neovascularization, and ideal tissue growth [12]. Ideally, oxygen-releasing materials must deliver an oxygen supply for 1–2 weeks, which is the range needed for vascularization post-implantation representing a critical period in which cells need oxygen, and it must be satisfied by an external supply [13].

Vascularization is followed by three stages. First, hypoxia will allow for angiogenesis, followed by hyperoxia, and finally, recovery when the tissue needs extra oxygen, but without producing radioactive species or pH changes to avoid inducing apoptosis to allow for complete vascularization [14]. One of the most critical parameters is the controlled release of oxygen, which means the amount of oxygen must not exceed the percentage of oxygen released because an excess of oxygen can inhibit vascularization, differentiation, and/or cause tissue damage. Calcium peroxide nanoparticles (CPO) are oxygen-releasing materials that can be loaded inside hydrogels because CPO have some better characteristics than other compounds, such as higher purity, more controllable oxygenation, and its initial supersaturation does not occur as in liquid peroxides. In the presence of a catalase enzyme, the chemical reaction entirely avoids the formation of reactive oxidant species (ROS), and previous research has verified the controlled and sustained release of oxygen by CPO [12].

In this context, this review collates relevant information from the literature concerning 3D hydrogel bioprinting for tissue regeneration, mainly for cartilage applications. Therefore, it has been divided into several sections to allow for a better understanding of the subject matter. It also includes descriptions of different bioprinting techniques and the classification of the different types of hydrogels and nanoparticles that could be used with scaffolds to allow for chondrocyte proliferation and hyaline tissue regeneration. Finally, the relationship between oxygen and cartilage tissue is discussed. In this review, we used the keywords “Biomaterials”, “Oxygen release”, “Cartilage”, and “Bioprinting” for the inclusion and exclusion of research articles according to relevance and impact factor.

## 2. Oxygen and Cartilage Tissue

An ample oxygen supply is essential during the tissue-forming process because oxygen is a prerequisite for cell proliferation and differentiation. Although articular cartilage cells have adapted for survival in a hypoxic environment, an adequate oxygen level is essential for their normal metabolic activity. Oxygen levels below 1% significantly alter chondrocyte activity inhibiting glucose uptake and cellular ribonucleic acid (RNA) synthesis. In addition, oxygen levels down to 1% cause cells to produce calcified cartilage [15].

Grimshaw and Mason also showed the positive role of oxygen in chondrocyte metabolism. They compared the comportment of bovine articular chondrocytes cultured in alginate beads for 7 days in cell culture medium maintained at different oxygen levels (<0.1, 5, 10, and 20%). The results demonstrated that articular chondrocytes must be cultured at oxygen levels between 5 to 10%, since such cells showed greater activity in this range [16].

**Figure 1:**
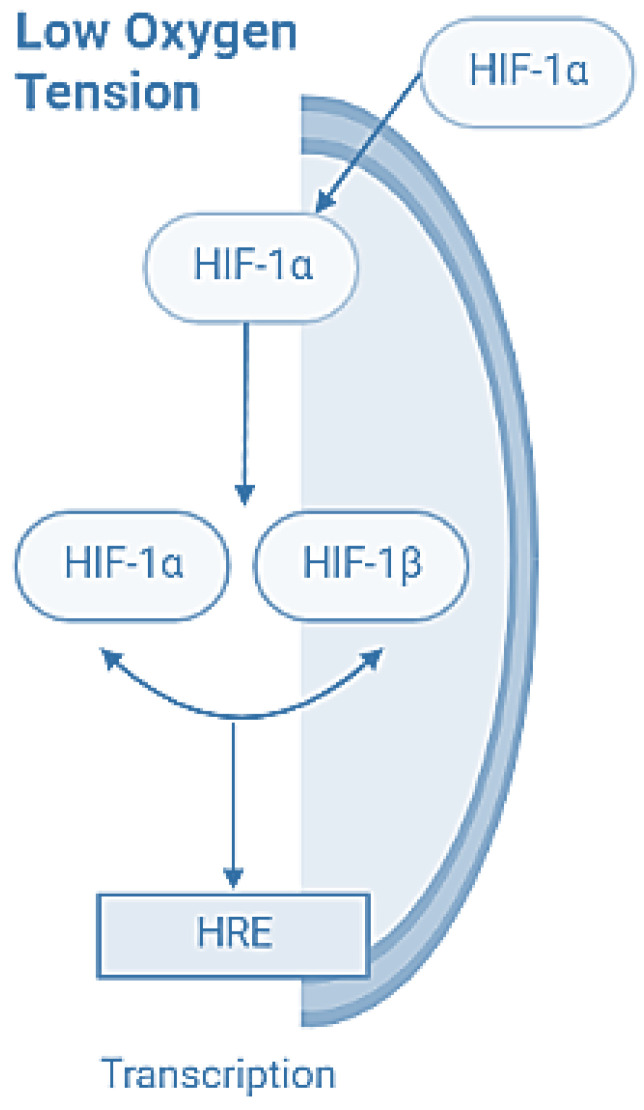
HIF-1α oxygen-sensing pathways.

Chondrocytes must adapt to a low oxygen tension environment to produce adenosine triphosphate (ATP) by substrate-level phosphorylation in glycolysis because glucose uptake, such as lactate production and glucose metabolism, is inhibited by less than 1%. This is why chondrocytes need at least some oxygen for their basal metabolic activity. There is evidence that chondrocytes express hypoxia-inducible factor 1α (HIF-1α); HIF-1α by low oxygen, translocates to the nucleus where it dimerizes with HIF-1β to form an active transcription factor. HIF-1α recognizes and binds to hypoxia response elements (HRE) for the promoters of target genes, including those involved in angiogenesis and cell proliferation, and regulates glycolysis (Figure 1) [17]. 

## 3. Advances in Oxygen Delivery from Biomaterials for Cartilage Tissue Engineering

**Figure 2:**
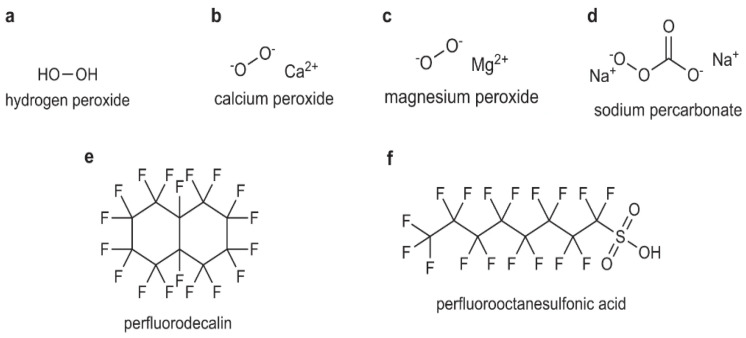
Chemical structures of oxygen-releasing elements: (**a**) hydrogen peroxide, (**b**) calcium peroxide, (**c**) magnesium peroxide, (**d**) sodium percarbonate, (**e**) perfluorodecalin, and (**f**) perfluorooctanesulfonic acid (PFOS) [5].

Oxygen-releasing smart materials can be created as carrier biomaterials to avoid a burst release of oxygen-inducing cytotoxicity and, therefore, allow oxygen release over a prolonged time sufficient to support cells during angiogenesis. This can be accomplished through the incorporation of peroxides of solids, liquids, or fluorination types, which are incorporated into constructs of different forms (such as microspheres, nanoparticles, films, electrospun nanofibers, electrosprayed materials, scaffolds, etc.) and in various materials, including polymers and ceramics; such approaches have been used in the past to control drug release [5,18,19]. Figure 2 shows the chemical structures of different oxygen-releasing elements.

Biomaterials with oxygen-releasing nanoparticles are considered smart materials with potential applications in the biomedical market, especially for cells and tissues with more metabolic activity, and as carrier materials for oxygen supplementation to maintain healthy tissues (e.g., cardiac, pancreas, muscle, cartilage, and skin) [20]. Various types of nanoparticles and their compounds, in combination with hydrogels, not only generate structural diversity, but also improve mechanical strength and responses to a plurality of stimuli [21]. 

### 3.1. Solid Inorganic Peroxides

Sodium, calcium and magnesium peroxides are the most used solid inorganic peroxides. Hydrolysis is the primary mechanism for oxygen release when these nano/microparticles interact with water, as shown in these equations [5,19,22]:

Calcium peroxide

(1) $$\mathrm{Ca}O_{2}\left(s\right)+2H_{2}O\to \mathrm{Ca}{\left(\mathrm{OH}\right)}_{2}\left(s\right)+H_{2}O_{2}$$

Magnesium peroxide

(2) $$\mathrm{Mg}O_{2}\left(s\right)+2H_{2}O\to \mathrm{Mg}{\left(\mathrm{OH}\right)}_{2}\left(s\right)+H_{2}O_{2}$$

Sodium percarbonate

(3) $$\left({\mathrm{Na}}_{2}{\mathrm{CO}}_{3}\right)*3H_{2}\to 4{\mathrm{Na}}^{+}+2{\mathrm{CO}}_{3}^{-2}+3H_{2}O_{2}$$

(4) $$2H_{2}O_{2}\to O_{2}+2H_{2}O$$

Generally, MgO_2_ has a faster reaction rate that, in turn, causes supersaturation. High saturation is not efficient because cell death occurs, as the oxygen rate delivery is insufficient to allow for cell proliferation and survival. This is what happens when perfluorocarbonates are used [9].

Calcium-based nanoparticles have already been used in different applications, including tissue regeneration, and allow for a sufficient amount of oxygen delivery with a sufficient reaction rate for cells to proliferate. This type of reaction in the presence of an enzyme (such as catalase) allows for the complete decomposition of the peroxide and does not cause secondary toxic products, such as H_2_O_2_ [18,23]. Table 1 summarizes some types of oxygen-generating composites, as well as their solubility coefficients and approximate oxygen-releasing amounts. 

### 3.2. Liquid Inorganic Peroxides

Some biomedical applications have used liquid peroxides to form oxygen-releasing particles. They present excellent solubility in an aqueous medium, as the water permits fast oxygen release. Their decomposition can be divided into oxygen and water. When the catalase enzyme of the human body is present in the liver, blood is transformed into water and oxygen. The decomposition follows these reactions [5]:

H_2_O_2_ (aq) + 2Fe^3+^(aq) → O_2_(g) + 2Fe_2_^+^(aq) + 2H^+^ (aq) (5)

H_2_O_2_ (aq) + 2Fe^3+^(aq) + 2 H^+^(aq) → 2 H_2_O(l) + 2Fe^2+^(aq) (6)

The reaction velocity is a problem when hydrogen peroxides are used, due to a significant reaction at the initial supersaturation of cells. Abdi et al. encapsulated H_2_O_2_ into poly lactic-co-glycol acid (PLGA), which was coated by a secondary layer composed of an alginate hydrogel with the catalase incorporated to allow for total decomposition of the hydrogen peroxide into oxygen and water, avoiding the generation of harmful radicals that influence cell viability in scaffolds, but release oxygen [24].

Choi et al. fabricated polymeric microspheres for sustained oxygen-release and developed a sponge by embedding microspheres into an alginate-based hydrogel that can supply oxygen for wound healing in vitro and in vivo. A double emulsion method was used with PLGA to form a porous oxygen-releasing hydrogel sponge (ORHS). The results showed that oxygen release induced neovascularization and cell proliferation, helping wound healing. On day 7, a complete formation of the skin layer was observed [25]. 

### 3.3. Methods and Kinetics of Released Oxygen

The release of oxygen from nanoparticles that are hybridized into polymeric matrices, as well as drug release, is controlled via diffusion, swelling of the hydrogel, reversible nanoparticle-polymer interactions, and degradation of labile covalent bonds, osmosis, and erosion [26,27]. 

The diffusion mechanism and matrix porosity play important roles. Other important parameters include the size of the nanoparticles in the hydrogels, where results have shown that a larger size of incorporated nanoparticles produces a lower release rate and increases insolubility. The release rate increases with insoluble or poorly soluble substances [28]. 

In most hydrogel matrices, nanoparticle release is controlled by the diffusion of the nanoparticles to the outside of the matrix. The release of nanoparticles is time-dependent; when time increases, the release of nanoparticles decreases. While the release rate decreases with time, there may be a saturation point where diffusion can no longer be controlled and is instead controlled by the dissolution of the nanoparticles in the presence of a liquid medium. This is called zero-order release kinetics [29].

For hydrophilic matrices, the main variables that affect the release are the type of the polymer, the nanoparticle/polymer ratio, the solubility of the nanoparticles, the size of the nanoparticles, and other hydrogel factors, such as the viscosity, compression force, incorporation, and distribution of nanoparticles in the matrices. The matrices in contact with an aqueous medium are rapidly hydrated and form the solid–liquid interface. When the liquid medium enters the hydrogel matrix, swelling, rearrangement, and relaxation of the polymer chains occur, increasing the volume causing the outermost layers to undergo an erosion process. The release of oxygen will occur in the transition from the crystalline state of the matrices, due to water penetrating the layers. In the case of solid peroxide nanoparticles, a chemical reaction occurs, and the oxygen molecules are released through the pores of the matrix [26,28]. 

The oxygen rate is essential to allow for vascularization into particular tissues where enough oxygen in cells is crucial for cellular survival in hypoxic conditions and to avoid inhibiting vascularization, cell differentiation, cell necrosis, or tissue damage from the excess release of oxygen. Thus, the control of oxygen release stems from four reasons: (1) oxidative damage to cells is caused by hyperoxia and by reactive oxygen species (ROS); (2) cell differentiation is affected by ROS; (3) the inflammatory process is produced by ROS, which acts as a mediator, and (4) adequate hypoxia stimulates vascular infiltration [30]. 

**Figure 3:**
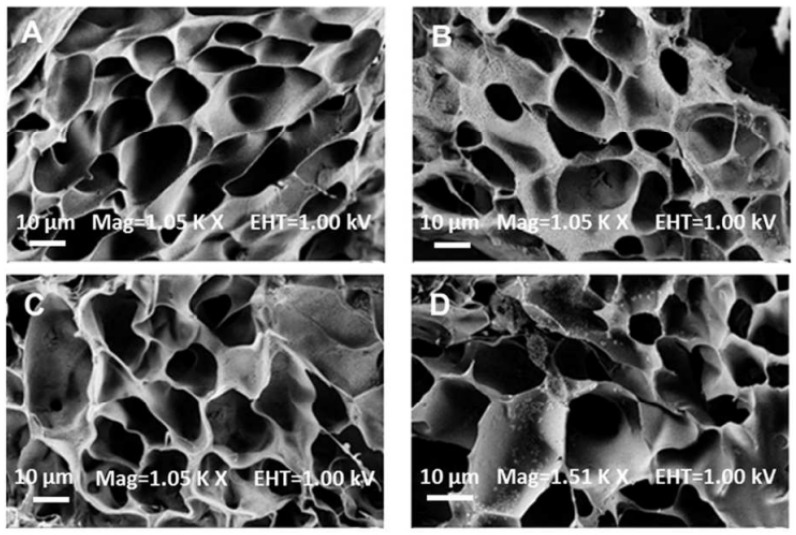
SEM images of GelMA/CPO composites with different concentrations of CPO: (**A**) 0% CPO, (**B**) 1% CPO, (**C**) 2% CPO, and (**D**) 3% CPO. High concentrations of CPO increased porosity and swelling [23]. Copyright 2017, American Chemical Society.

Nanoparticles composed of peroxides are preferred because they respond successfully in an aqueous medium in combination with hydrogels [18]. The inclusion of calcium nanoparticles also helps improve porosity and swelling. Alemdar et al. used different concentrations of CPO at 1, 2, and 3% *w*/*w* in GelMA hydrogels. This study showed that the porosity and swelling were directly related (Figure 3). The swelling ratio increased from ∼17 ± 0.8 to ∼27 ± 0.7 for the GelMA hydrogels upon the incorporation of 3% CPO, and cell survival was 80% at 3% CPO at day 5, compared to the control, which was 60% [23]. 

**Figure 4:**
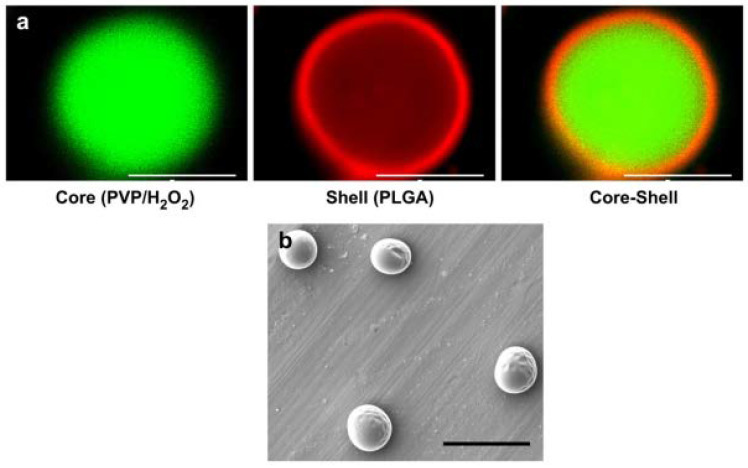
Confocal and SEM images of VP/H_2_O_2_ core-shell oxygen-releasing nanoparticles designed to encapsulate cardiosphere-derived cells (CDCs): (**a**) confocal images of a polymer core of PVP/H_2_O_2_, scale bar = 5 μm, and (**b**) SEM image of oxygen-releasing microspheres. Scale bar = 10 μm. Reprinted from [13]. Copyright 2012, with permission from Elsevier.

Another study by Li et al. encapsulated hydrogen peroxide and poly(2-vinlypyrridione) (PVP) into PLGA to form microparticles, which were loaded into a thermosensitive hydrogel from hydroxyethyl methacrylate oligo (hydroxybutyrate), N-isopropylacrylamide (NIPAAm), and acrylic acid (AAC) (Figure 4). Cardiosphere-derived cells (CDCs) were also loaded to form a hybrid hydrogel. The results showed a homogeneous distribution of cells in the 3D structure, and a significant increase in cell viability with the oxygen-generating hydrogels was observed when placed in a hypoxic environment for up to 2 weeks. Therefore, this can serve as a biomaterial for cardiac tissue [13]. 

## 4. Applications of Scaffolds That Deliver Oxygen for Cartilage Tissue Regeneration

### 4.1. Bioprinting as an Alternative to Creating Smart Scaffolds

Three-dimensional bioprinting offers new approaches to solving traditional tissue engineering problems, such as the absence of controlled and functional histoarchitectures [31], as well as a lack of ability to customize structures and shapes [32]. Other 3D bioprinting research includes cells and biomolecules seeded into a structure that acts as a scaffold with a porous structure similar to the ECM [33], cancer models [34], drug tests, biomimetic tissue models [35], and also to develop living tissue constructs in vitro [19]. 

Bioprinting involves three steps: preprocessing, processing, and post-processing [36,37]. In preprocessing, a design is created by fusing the desired model with computer-aided design (CAD). Alternatively, the design can also be generated with a blueprint of tissues and organs using image techniques, such as computed tomography (CT) or magnetic resonance imaging (MRI) [38]. The printing and post-printing are equivalent to the simultaneous deposition of cells and biomaterials using deposition techniques [34,39].

The main 3D bioprinting techniques are divided into three categories: (i) Extrusion-based bioprinting techniques that use mechanical forces to extrude the material through a hole [38,40]. (ii) Inkjet-based techniques that eject ink drops from picoliters of the material onto a substrate [41,42]; and (iii) Laser-based techniques where a laser is used to cure photopolymers or induce material injection [38,43]. 

**Figure 5:**
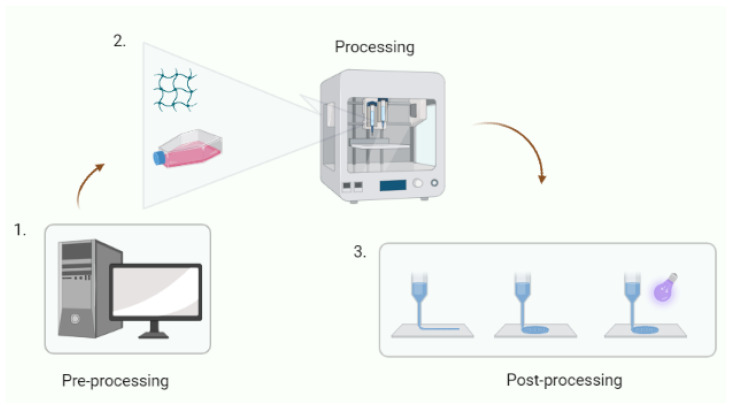
Schematic diagram showing the steps for bioprinting and 3D bioprinting. 1. Pre-processing, in this step, the structure is designed with software. 2. Processing is the second step, where the design is printed using pneumatic pumps. This step can combine cells with biomaterials (Reprinted from [49]) 3. This is the final step, where extrusion techniques can be used for printing material, pneumatic pressure, and finally, photo-cross-linking using light UV, depending on the material and application (adapted from [49], copyright 2018, with permission from Elsevier).

In the extrusion of the material through a hole, mechanical forces are used, and generally, two different configurations are used in bioprinting based on the extrusion of the material: the mechanical (1) piston motor and (2) pneumatic pumps [38,44,45]. These types of extrusion-based techniques work best with materials with slimming properties, such as alginate [46], gelatin [47], and gelatin methacrylamide [48], amongst others. Figure 5 shows a schematic of bioprinting and 3D bioprinting approaches.

### 4.2. Bioprinting Hydrogels to Deliver Oxygen for Cartilage Tissue

Hydrogels are polymeric, hydrophilic, and have three-dimensional networks. This special type of material can absorb large amounts of water or fluids, in addition to having excellent biocompatibility [34], and can be tuned to a particular application, including drug delivery and tissue repair. Hydrogel studies have been increasing over the last decade, and there has been three times the number of publications between 2016 and 2020, representing an increase of up to 1.4 times; this is about 74% of the number of studies on cartilage hydrogels [50]. These hydrogel properties have turned out to be highly promising for medical applications for drug delivery and tissue regeneration. However, the lack of biodegradability of polymeric materials has been problematic in the past. However, some natural and synthetic polymers have been used as a basis for scaffold construction that promotes angiogenesis in tissue engineering, such as collagen, gelatin, chitosan, silk, and fibroin, due to the high flexibility present [51]. Hydrogels that are hybrids with synthetic and natural polymers have been shown to be better for encapsulating cells [20]. 

#### 4.2.1. Natural Polymers

Natural polymers are cheaper and often mimic the extracellular matrix (ECM), providing better biocompatibility and adhesion with cells. However, the variation in the quality of naturally occurring polymers and the portions extracted from different sources are obstacles to reproducibility [51]. Natural hydrogels exhibit some limitations, as they do not present strong mechanical properties and cannot easily be controlled. Engineered scaffolds have been used as protein-based materials, such as hyaluronan, collagen, gelatin, and fibrin, because they present advantages in extracellular environments [52].

Hyaluronan can provide cells a 3D environment that is very similar to the natural matrix because they are components of the extracellular matrix. La Gatta et al. synthesized sponge-based scaffolds by lysine methyl-ester cross-linking at different amounts and hyaluronan to obtain materials that closely resemble the elements in physiological cellular environments. Their results demonstrated that the water uptake and mechanical, morphological, and stability properties were comparable or superior to hyaluronan scaffolds, and the chondrocytes cultured were maintained for 3 weeks. Thus, these are promising hydrogels for cartilage repair [53]. 

Collagen is another natural material with outstanding properties, such as low antigenicity, biodegradability, biocompatibility, and cell adaptation, which allows it to be commonly used in biology and medicine. However, natural collagen’s degradation rate and mechanical stability are insufficient to fulfill the requirements of tissue engineering; therefore, collagen needs to be modified, and its properties cross-linked [54].

Zhenhui et al. conjugated biocompatible carbon dot nanoparticles (CP NPs) onto collagen through a natural product cross-linker (genipin) to prepare an injectable hydrogel. They demonstrated that increased stiffness, due to the cross-linking, presented a 21-fold higher compression modulus and a 39.3% lower degradation rate than pure collagen, and the hydrogel increased cell proliferation by 205.1% on day 21 [55].

Fibrin is also a material used as a biomaterial for tissue engineering. It has been investigated as a vehicle for drug delivery. However, fibrin is not chondro-permissive and clearly requires further functionalization to be a versatile injection hydrogel system used in cartilage repair therapies [56]. 

Gelatin is another hydrogel currently being used as a biomaterial with advantages in biocompatibility, as well as biodegradability, and it is inexpensive. Gelatin is extracted from collagen hydrolysis and can absorb water 5–10 times its weight. However, despite the versatility of gelatin within different biomaterial fields, it has weak mechanical stability and durability; therefore, gelatin needs to be polymerized with anhydride groups to obtain GelMA hydrogels with cross-linked chains for the free movement of cells within the matrix, and through the use of a photo-cross-linking agent, the degradation properties are improved [57].

#### 4.2.2. Synthetic Polymers

Synthetic polymers present acceptable processing flexibility and do not have immunological concerns, compared with natural polymers [58]. Cartilage tissue engineering has used a variety of synthetic polymers, such as polyglycolic acid (PGA), polylactic acid (PLA), poly (ethylene glycol) (PEG), poly (vinyl alcohol) (PVA) polydioxanone, and methacryloyl gelatin hydrogels (GelMA), due to their mechanical, porosity, swelling, and lubricating properties, similar to natural cartilage [59,60,61]. Some properties of synthetic biodegradable polymers are shown in Table 2.

Cui et al. used a poly (ethylene glycol) (PEG)-based hydrogel implant loaded with chondrocytes fabricated using extrusion bioprinting and showed a high integration with adjacent cartilage tissue [64]. On the other hand, Schuurman et al. used GelMA in combination with hyaluronic acid (HA) to increase the viscosity of the precursor material and demonstrated a printing fidelity and chondrocyte viability over photo-cross-linking [65]. Meanwhile, in another research, Galarraga et al. used norbornene-modified hyaluronic acid (NorHA) and cross-linking over visible light, and the printing process allowed for favorable cytocompatibility with high cell viability, a homogenous distribution of mesenchymal stromal cells (MSCs), and over 56 days of culture in chondrogenic media [66]. 

Thus, the main focus and key to the future of cartilage regeneration deals with the mechanical properties of the materials; a high resistance will promote mechanical stability of a joint, and high-water content will maintain a lubricious surface. In addition, bioactivity is also required to sustain cell proliferation and migration; the materials that make up the scaffold should retain a capacity for biodegradation and a possibility to remodel as new cartilage, considering that for cartilage to totally regenerate, it will require a longer time period, unlike other tissues [67]. 

## 5. Future Developments: Bioprinting of GelMA Hydrogels

The previous subjects have already discussed different materials used for cartilage tissue regeneration, either natural or synthetic, or both. Synthetic polymers are an attractive choice for scaffold formation because of their reproducible properties, controllable degradation, mechanical properties, and high purity, but due to their low bioactivity and biocompatibility, their clinical practice is limited, that combine the advantages of natural and synthetic hydrogels [68]. Therefore, biopolymers of natural origin have been researched and used to develop scaffolds with similar components to the cartilage ECM [69]. 

**Figure 6:**
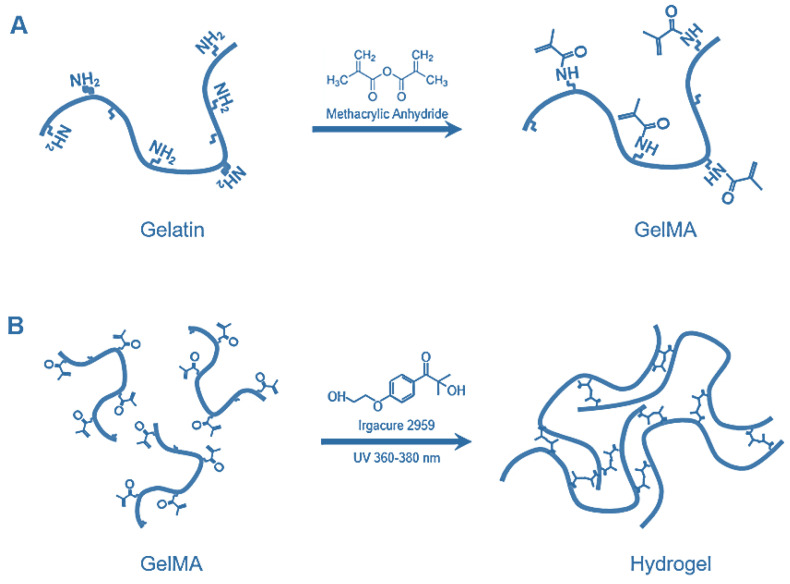
Synthesis and fabrication of photo-cross-linked gelatin methacryloyl (GelMA). (**A**) Gelatin is reacted with methacrylic anhydride (MA) to introduce a methacryloyl substitution group on the amino acid residue reactive amine and hydroxyl groups. (**B**) Photo-cross-linked GelMA.

GelMA is derived from gelatin (porcine or fish) and presents essential characteristics, such as lower immunogenicity, compared to its precursor (denatured collagen) [70]. Arginine-glycine-aspartic acid (RGD) is the bioactive part of gelatin, which stimulates the adhesion and growth of cells, and a metalloproteinase (MMP) matrix can be used for cell remodeling. Gelatin modification is obtained when the gelatin reacts with methacrylic anhydride and is optically cross-linked in the presence of photo-initiators [70,71]. Figure 6 shows the synthesis of GelMA hydrogels.

Mechanical strength, as well as porosity, degradation, and swelling, are critical properties for scaffold materials used to repair cartilage tissue. The mechanical properties of GelMA hydrogels are essential to allow for encapsulated cells. This property can be adjusted by changing the degree of the substitution of methacrylic anhydride, photo-cross-linking time, and the concentration of GelMA.

Bioprinting structures of GelMA have been a challenge for many researchers, due to the difficulty in printing time and due to viscosity. GelMA requires lower temperatures than other materials to increase viscosity and facilitate printability. GelMA/gelatin has been previously reported by Yin et al., which is a dual-combination material in which a better percentage for printing was found to be 5% (*w*/*v*) GelMA with 8% (*w*/*v*) gelatin forming a two-step cross-linking process combining reversible and irreversible thermal and photo-cross-linking; in vitro viability was more than 90% [48]. Initially, the requirements for bioprinting GelMA hydrogel structures loaded with smart particles that release oxygen included: adequate viscosity and faster cross-linking printable at lower temperatures to avoid cell death, including using a sacrificial material to increase the viscosity. Yin et al. used 8% (w/t) gelatin with 5% GelMA as better percentages to obtain a printed hydrogel structure [48]. 

Zhao et al. showed that the mechanical strength increased with GelMA concentration, but the swelling and degradation decreased [72] (some properties are shown in Table 3). Celikkin et al. obtained 80 ± 10% porosity for 5% GelMA scaffolds and confirmed that the porosity decreased to 60 ± 10% when the GelMA concentration increased to 10% *w*/*v* [73]. 

Schuurman et al. printed structures of GelMA with HA with an effective swelling ratio dependent upon UV exposure radiation. The swelling ratio of hydrogels decreased by 60% after 5 min exposure. They confirmed negligible swelling after at least 25 min of UV exposure and demonstrated that the gels obtained about two-thirds of their maximum modulus after approximately 10 min of UV exposure [65]. 

Hence, bioprinting polymeric constructs loaded with cells are favorable for parameters, such as first layer formation, viscosity, shear thinning, and cross-linking mechanisms. The first layer is the main parameter for which the hydrogel is printed, due to the structure’s maintenance, avoiding flattening the painted hydrogel precursor solution. The second parameter discussed is viscosity, which is directly related to a hydrogel being favorably or unfavorably printed [76]. A third parameter entails shear thinning, due to viscosity decreasing as the shear rate increases simultaneously, which favors embedded cell survival. Finally, cross-linking of the materials is mainly required because fast gelation allows for good printing and shapes fidelity when the extruded polymer is needed. Hence, high viscosity is essential, since it is inappropriate for tissues and impedes cell survival and proliferation. Currently, ionic, photo, and thermal cross-linking are bioprinting’s most commonly used cross-linking mechanisms [76] (Table 4).

Due to the characteristics of 3D printing and high impact, which is being used and applied in some sciences, such as engineering and medical sciences, and to serve as a medium for making new structures, GelMA has primarily been studied for bone and skin applications [84,85]. The results have shown that it is highly biocompatible with low toxicity. In addition, GelMA can serve as a carrier material for delivering sufficient quantities of oxygen to avoid cellular death and allow for cellular proliferation and regeneration of the cartilage tissue [71]. Specifically, Alemdar et al. reported that structures synthesized with GelMA and CPO help tissue regeneration, due to improved cellular survival [23]. Cartilage tissue presents a significant tissue engineering problem due to its lack of oxygen and poor tissue regeneration. Three-dimensional bioprinting offers a novel method with the possibility of oxygen delivery for higher cell survival for more chondrocyte growth and cartilage tissue regeneration.

## 6. Conclusions

Bioprinting has been a focus recently due to many advantages, including placing cells in hydrogels and simulating the behavior of the tissues and organs to be regenerated. Synthetic polymeric materials with similar characteristics mimic the native extracellular matrix (ECM), such as porosity, biocompatibility, and biodegradability. They can be loaded with nanoparticles (CPO, PFC, SPO, and others) acting as oxygen-releasing biomaterials that respond to external stimulation to repair damaged tissue for cartilage engineering applications by cell ingrowth. They have been demonstrated to be good candidates for use in in vitro and in vivo assays.

Additional research is still needed concerning biomaterials for cartilage tissue repair. The development of smart biomaterials for cartilage tissue repair with controlled oxygen-releasing properties would help tissue engineering. It would be innovative to replace the commonly used tissue regeneration techniques, such as debridement and lavage, microfracture, and autografts (cell and tissue transplantation). These types of therapies have been shown to repair cartilage defects effectively. However, there are some limitations, such as a lack of integration with healthy cartilage, little existing nutrients, and the formation of fibrous tissue, instead of hyaline cartilage, that present morphologies and functions consistent with unsuccessful cartilage tissue growth. It is possible that incorporating such scaffolds with oxygen-releasing nanoparticles could, in hypoxic environments, generate enough oxygen for the survival of chondrocytes.

## Figures and Tables

**Table 1:** The types of oxygen-generating composites and their solubility coefficients and oxygen-release to tissue regeneration are summarized [5].

Compound	Solubility Coefficient	Amount of Oxygen Release
Calcium peroxide	1.65 g/L at 20 °C	22 ± 3.3 mg/L
Magnesium peroxide	0.086 g/L at 18 °C	44.38 mg/L
Sodium percarbonate	120 g/L at 20 °C	40 mg per 100 mL of O_2_, i.e., 57.16 mg per 100 mL O_2_

**Table 2:** Properties of synthetic biodegradable polymers for cartilage tissue engineering.

Polymers	Molecular Formula	Melting Point	Glass Transition Temperature	Fundamental Chemical Structure	Oxygen-Releasing Properties
Polyglycolic acid (PGA)	(C_2_H_2_O_2_)n	225–230 °C	35–40 °C	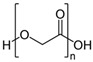	None.
Polylactic acid (PLA)	(C_3_H_4_O_2_)n	150–160 °C	60–65 °C	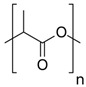	Using PLA has limited applicability for slow oxygen release [62].
Poly (lactic-co-glycolic acid) (PLGA)	-	It depends on the percent composition (PLA, PGA)	40–60 °C	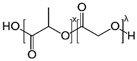	Over 10 days, oxygen is slowly released [63].

**Table 3:** Synthesis of hydrogels with desirable characteristics.

Regulating Variables	Effects on Mechanical Properties	Cell Types	Results	Ref
Concentration of GelMA	Higher compressive modulus and lower swelling properties.	Bone marrow stromal cells (mBMSCs).	At 10% (*w*/*v*) GelMA hydrogels, 60 ± 10% of porosity and with an average pore size: 250 ± 65 mm.	[73]
Photo-exposure time	Cell viability: decreased with increasing exposure duration (5–20 s).	Odontoblast cells (OD21 cells).	Cell viability decreased at 60% after 24 h at 20 s of photo-cross-linking.	[74].
Degree of methacrylate degradation	Increasing the degree of substitution in GelMA increased the storage modulus of the resulting hydrogel.	Cellulosaurus cells (Huh-7.5 cells).	High degree of methacrylate substitution promoted cell extrusion from 67.6 to 1.9 kPa to 94.9% and 14.8% DS, respectively.	[75]
Oxygen-releasing properties	CSPs in GelMA under hypoxia underwent significant cell death, with as little as ∼45% surviving cells. In remarkable contrast, CPO-GelMA demonstrated up to ∼80% cell survival rates.	Cardiac side population cells (CSPs).	3% of CPO was able to raise the oxygen tension to ∼17% after 1 day, ∼13% after 3 days, and ∼8% after 5 days.	[23]

**Table 4:** Cross-linking types for some materials used in cartilage tissue regeneration.

Materials	Cross-linking	Advantages	Disadvantages	Encapsulated Cells	References in Bioprinting
Collagen	pH cross-linking (7–7.4) at 37 °C or thermal cross-linking	Biocompatibility, high cell adhesion, promotes cell proliferation to serve as a signal transducer, high printability.	Low gelation rate, poor mechanical properties, and stability.	BMSC fibroblasts chondrocytes	[77,78]
Fibrin	Enzymatic cross-linking	Biocompatibility, high cell adhesion, rapid gelation.	Limited printability and poor mechanical properties.	BMSC chondrocytes	[79,80]
Pluronic F127	Irreversible thermal cross-linking at 24–37 °C	Biocompatibility, high printability, supports cell viability.	Weak stability and mechanical properties, fast degradation, slow gelation.	BMSC fibroblasts	[81]
Poly(ethylene glycol)	Thermal cross-linking	Biocompatibility, supports cell viability, and can be easily modified with various functional groups.	Poor cellular adhesion and low cell proliferation rate.	BMSC chondrocytes	[82]
GelMA	UV cross-linking	Biocompatibility, can be modified, and low toxicity.	Low viscosity, needs low temperature.	Chondrocyte liver cells MSCs	[83]

## Data Availability

Not applicable.
